# Influence of ionizing radiation and cell density on the kinetics of autocrine destruction and intercellular induction of apoptosis in precancerous cells

**DOI:** 10.1038/s41598-022-11253-1

**Published:** 2022-05-03

**Authors:** Abdelrazek B. Abdelrazzak, Peter O’Neill, Mark A. Hill

**Affiliations:** 1 Spectroscopy Department, Physics Research Institute, National Research Centre, Cairo, 12622 Egypt; 2 MRC Oxford Institute for Radiation Oncology, University of Oxford, Gray Laboratories, ORCRB, Roosevelt Drive, Oxford, OX3 7DQ UK

**Keywords:** Biophysics, Cancer, Biological physics, Apoptosis, Extracellular signalling molecules, Growth factor signalling, Stress signalling

## Abstract

Intercellular induction of apoptosis (IIA) represents a well-defined signaling model by which precancerous cells are selectively eradicated through reactive oxygen/nitrogen species and cytokine signaling from neighbour normal cells. Previously, we demonstrated that the IIA process could be enhanced by exposure of normal cells to very low doses of ionizing radiation as a result of perturbing the intercellular signaling. In this study, we investigate the kinetic behaviour of both autocrine destruction (AD) and IIA as a function of cell density of both precancerous and normal cells using an insert co-culture system and how exposure of normal cells to ionizing radiation influence the kinetics of apoptosis induction in precancerous cells. Increasing the seeding density of transformed cells shifts the kinetics of AD towards earlier times with the response plateauing only at high seeding densities. Likewise, when co-culturing precancerous cells with normal cells, increasing the seeding density of either normal or precancerous cells also shifts the kinetics of IIA response towards earlier times and plateau only at higher seeding densities. Irradiation of normal cells prior to co-culture further enhances the kinetics of IIA response, with the degree of enhancement dependent on the relative cell densities. These results demonstrate the pivotal role of the cell seeding density of normal and precancerous cells in modulating both AD and IIA. These results further support the proposition that ionizing radiation could result in an enhancement in the rate of removal of precancerous cells through the IIA process.

## Introduction

Intercellular induction of apoptosis (IIA) represents a well-defined signaling model by which precancerous cells are selectively eradicated at an early stage of carcinogenesis through signaling from neighbouring normal cells^1–3^. Previous studies^2,4,5^ have demonstrated that this IIA process could be enhanced by exposure to very low doses of low- or high-LET ionizing radiation as a result of perturbing the balance of intercellular signaling, with subsequent plateauing at relatively low doses.

**Figure 1:**
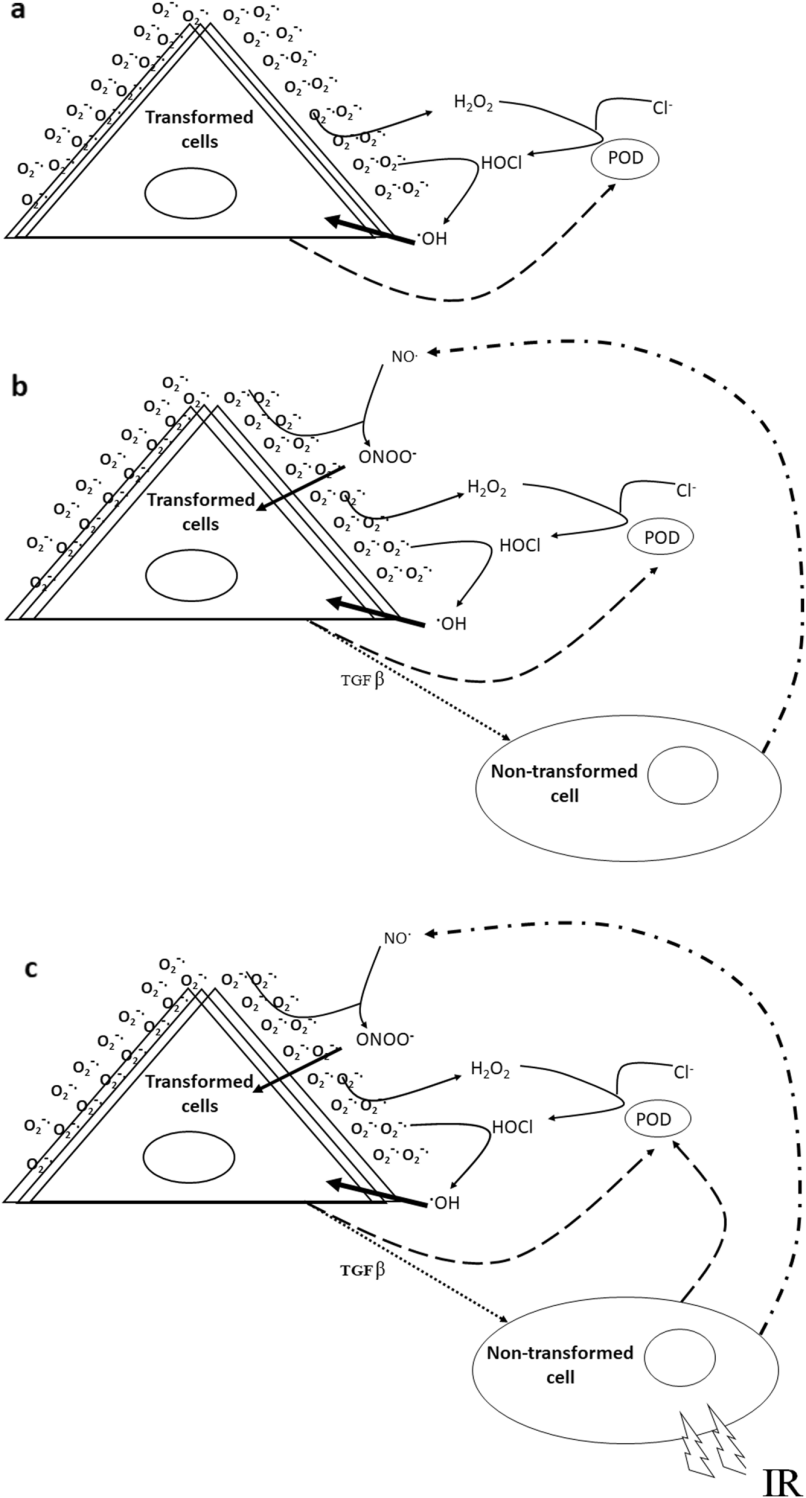
The mechanisms of AD and IIA. The mechanisms of apoptosis induction in 208F*src3* transformed cells by (**a**) autocrine destruction AD, (**b**) Intercellular induction of apoptosis IIA and (**c**) Radiation-induced intercellular induction of apoptosis. In the absence of non-transformed 208F cells, 208F*src3* transformed cells at sufficient densities undergo autocrine destruction through POD/HOCl pathway. In the presence of non-transformed 208F cells, 208F*src3* transformed cells experience additional apoptosis signaling through the NO^**·**^/ONOO^−^ pathway. Irradiation of non-transformed 208F cells stimulates the intercellular POD/HOCl apoptosis pathway between irradiated non-transformed and transformed cells, in addition to augmenting the pre-existing NO^**·**^/ONOO^−^ pathway, resulting in an overall enhancement in apoptosis induction in transformed 208F*src3* cells^15^.

Precancerous (transformed) cells overexpressing src oncogene, src-transformed rat fibroblast cell lines (208F*src3*), are phenotypically characterized by extracellular sustained production of superoxide anions (O_2_^−**·**^) in close vicinity to the cell membrane by membrane-bound NADPH oxidase. At sufficient cell concentrations, superoxide anions (O_2_^−·^) dismutate to produce hydrogen peroxide (H_2_O_2_). Transformed cells excrete peroxidase (POD), which interacts with H_2_O_2_ in the presence of chloride ions (Cl^−^) to produce hypochlorous acid (HOCl) in close proximity of the cell membrane. As a consequence, HOCl may interact with O_2_^−·^ to produce site-specific hydroxyl radical (^·^OH) close to the cell membrane to induce apoptosis (referred to as POD/HOCl pathway). This process is termed autocrine destruction (AD) (Fig. 1a).

If *src*-transformed cells are co-cultured with non-transformed 208F rat fibroblast cells, additional processes may occur whereby O_2_^−·^ derived from, and in close proximity to, the transformed cells interact with nitric oxide radical (NO^·^), produced by non-transformed cells, to form apoptosis-inducing peroxynitrite (ONOO^−^) close to the transformed cells. This pathway is referred to as NO^·^/ONOO^−^ pathway (Fig. 1b), whereby apoptosis is induced solely in the transformed cells through intercellular induction of apoptotic signaling^6,7^. In contrast to transformed cells, tumour cells are resistant to both AD and IIA due to acquired resistance against intercellular ROS-mediated apoptosis induction through expression of membrane-associated catalase, which renders them H_2_O_2_ resistant^8–10^.

In both AD and IIA mechanisms, O_2_^−·^ levels represent the major limiting factor, since its concentration at the cell membrane of transformed cells is crucial for both the production of hydrogen peroxide, involved in the POD/HOCl pathway and the production of ONOO^−^ as part of the NO^·^/ONOO^−^ pathway, and therefore the susceptibility of transformed cells to apoptotic signalling^11^. Additionally, O_2_^−· ^has a very short range of a few μm^12^, emphasizing that only O_2_^−·^, formed in close proximity of the transformed cells, results in the high selectivity of the IIA mechanism. Therefore, overexpression of the *src* gene and expression of the *ras* oncogene, which upregulates NOX1 transcription leading to generation of O_2_^−·^ at the plasma membrane, determine the sensitivity of transformed cells to AD and IIA signalling^13,14^. Irradiation of non-transformed cells, prior to co-culture with transformed cells perturbs IIA signalling by stimulating the non-transformed cells to produce POD and increase the production of NO^·^ (Fig. 1c)^15^. In addition, irradiated non-transformed cells stimulate neighbour non-irradiated non-transformed cells, resulting in an overall enhancement in apoptosis induction in transformed cells^2,4,5^. Previous data suggest that TGF-β production, or activation, plays a major role in the enhancement of IIA, and in particular following exposure to ionizing radiation^1,2^.

The current available data suggest that for early carcinogenesis of small pre-neoplastic lesions composed of a few transformed cells surrounded in close contact by a sea of healthy cells, IIA should be efficient at removing or reducing the number of transformed cells. Furthermore, very low doses of radiation have the potential of increasing the rate of their removal. The magnitude of removal of transformed cells through either AD or IIA is dependent on the levels of signalling molecules involved in both the POD/HOCl and NO^·^/ONOO^−^ pathways. The amplitude of the signals is reliant on both the density and the stimulation of both transformed and non-transformed cells. Likewise, time plays a pivotal role in AD and IIA^1,2,15^. The temporal features of AD and IIA responses suggest that sufficient time is needed for the response to be induced^16^ and under certain conditions to saturate. The variation of the AD and IIA kinetics with time, have been previously described for a single seeding density for either cell population^1,2,5^. To date, it is as yet not clear how varying the relative densities of co-cultured transformed and non-transformed cells influence the AD and IIA responses.

A previous study based on theoretical modelling of AD and IIA responses have predicted the behaviour of IIA response^17^. Under conditions similar to those described experimentally (low density of transformed cells relative to the high density of normal cells), a decrease in IIA response 24 h post co-culture and therefore a potential increase in carcinogenic risk, due to a highly non-linear behaviour to IIA signalling, was predicted. According to the theoretical predictions, for high concentrations of O_2_^−·^ at the cell membrane of transformed cells (which corresponds to a high density of transformed cells), O_2_^−·^ migrates away from the cell membrane of transformed cells prior to reaction with NO^·^ and, as a consequence, would therefore render the NO^·^/ONOO^−^ pathway ineffective in inducing apoptosis in transformed cells, as the apoptosis-inducing ONOO^−^ would be formed distal from the transformed cells.

The aim of this study is to explore experimentally how the kinetic behaviour of AD and IIA responses under various co-culture conditions of transformed and non-transformed rat fibroblast cells, in the absence and presence of radiation, are modulated to gain a better understanding of the contribution of AD and IIA in the signaling mechanisms. The variation of the temporal response of both AD and IIA kinetics has been explored as a function of cell density of both transformed and non-transformed cells. In particular, the range of cell densities explored covered the region in which the theoretical modelling^17^ had predicted a decrease rather than an increase in the observed rate of apoptosis in the transformed cells. In addition, we have investigated further the perturbation of both responses by ionizing radiation.

## Results

For the purpose of this study, 208F*src3* transformed cells were seeded at various densities (10, 50, 100,110, 250, 500 and 1000 cells/mm^2^) and cultured for 94 h in the absence (negative control group) or presence of a fixed seeding density of non-transformed 208F cells (100 or 1000 cells/mm^2^), either sham or irradiated with 0.5 Gy γ-radiation. Further, 208F*src3* cells at a fixed seeding density (110 cells/mm^2^) were cultured either alone or in the presence of different seeding densities of 208F cells (100, 500, 1000 and 5000 cells/mm^2^) either sham- or 0.5 Gy γ-irradiated for comparison with the theoretical predictions^16^.

### **IIA response in 208F***src3***cells seeded at varying seeding densities in absence or presence of 100 cells/mm**^**2**^** of 208F cells either sham or 0.5 Gy γ-irradiated**

Firstly, we investigated the effect of co-culturing a fixed, relatively low seeding density of 208F cells (100 cells/mm^2^), either sham or 0.5 Gy γ-irradiated, with varying seeding densities (10, 50, 100, 110, 250, 500 and 1000 cells/mm^2^) of 208F*src3* cells on the IIA response. In parallel, the same seeding densities of 208F*src3* cells were cultured in the absence of 208F cells, to establish the level of apoptosis induction in 208F*src3* cells as a result of autocrine destruction.

**Figure 2:**
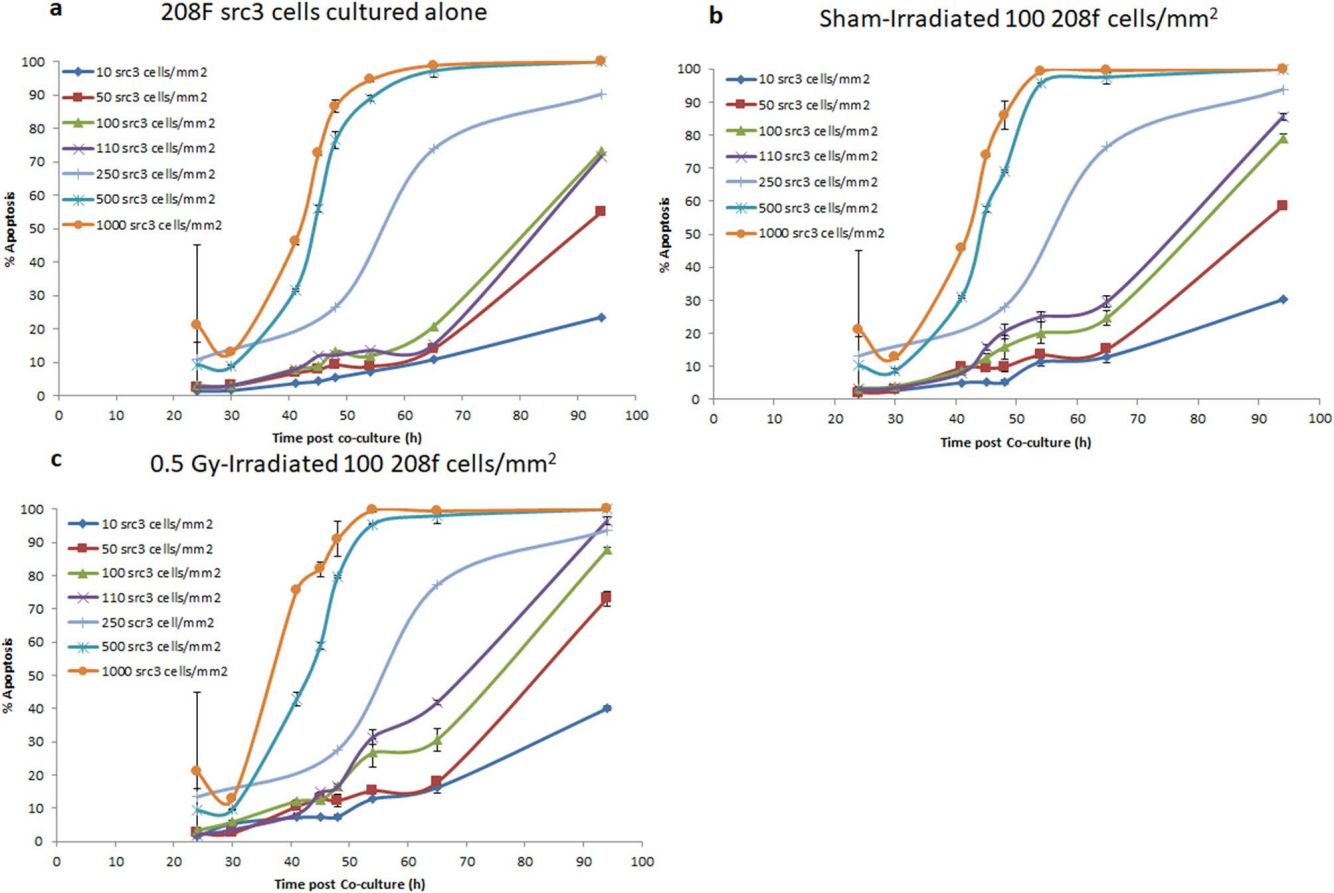
Low density 208F cells and varying density of 208F*src3* cells. Apoptosis induction in transformed 208F*src3* cells seeded at seeding densities 10, 50, 100,110, 250, 500 and 1000 cells/mm^2^ cultured in the absence (**a**) or presence of sham- (**b**) or 0.5 Gy γ-irradiated (**c**) non-transformed 208F cells at a seeding density of 100 cells/mm^2^. Each data point is a mean value derived from three independent experiments. Error bars represent the standard deviation derived from three independent experiments.

Figure 2a shows that apoptosis induction in unirradiated 208F*src3* cells (negative control group) cultured in the absence of 208F cells is dependent on the initial seeding density. For the two highest seeding densities of 208F*src3* cells (500 and 1000 cells/mm^2^), the onset of apoptosis induction occurs as early as 24 h post culture. The two densities then follow similar apoptosis induction kinetics with differences in the response behaviour seen mainly at < 65 h, after which time the apoptosis levels for both densities saturate (all cells are apoptotic). Similarly, for a seeding density of 250 cells/mm^2^, apoptosis induction is notable as early as 24 h post-culture, reaching a level of ~ 90% at 94 h. At lower seeding densities 10, 50, 100 and 110 cells/mm^2^, a delay in apoptosis induction was seen with no significant differences seen between the four densities up to 65 h. Subsequently, the medium densities (100 and 110 cells/mm^2^) show apoptosis levels higher than those of the lowest densities used of 10 and 50 cells/mm^2^. At 94 h, the apoptosis levels are 71, 71, 54 and 23% for the cell densities of 110, 100, 50 and 10 cells/mm^2^, respectively.

When sham-irradiated 208F cells, seeded at a density of 100 cells/mm^2^, were co-cultured with unirradiated 208F*src3* cells seeded at a density of 10, 50, 100, 110, 250, 500 and 1000 cells/mm^2^ (Fig. 2b and Supplementary Fig. 1), no significant difference in the IIA response was seen when compared with the negative control group, except for the density110 cells/mm^2^, when a significant increase (*p* < 0.05) in the IIA response was observed from 54 h onwards. On the contrary, 0.5 Gy γ-irradiation of 208F cells (seeded at a density of 100 cells/mm^2^) prior to co-culture with 208F*src3* cells results in an increase in the rate of induction of apoptosis in the unirradiated 208F*src3* cells, when compared with that of the sham-irradiated group. The radiation-induced IIA response enhancement was cell density-dependent. For 208F*src3* cells seeded at densities of 10, 50 and 100 cells/mm^2^, a significant enhancement in the IIA response following irradiation (*p* < 0.05) was only seen at the late time point of 94 h. For the seeding density of 110 cells/mm^2^, significant enhancement of IIA response was recorded at the earlier time point of 65 h and was sustained until the last time point measured at 94 h. For seeding densities of 250 and 500 cells/mm^2^, no significant change was recorded. However, for the higher seeding density of 1000 cells/mm^2^, a significant enhancement of the IIA response (*p* < 0.05) was only seen in the time range 30–45 h post co-culture, after which the response saturates.

### **IIA response in 208Fsrc3 cells seeded at varying seeding densities in the absence or presence of 1000 cells/mm**^**2**^** of 208F cells either sham or 0.5 Gy γ-irradiated**

The effect of increasing the initial seeding density of 208F cells to 1000 cells/mm^2^ on the IIA response was explored using various seeding densities of 208F*src3* cells (10, 50, 100, 110, 250, 500 and 1000 cells/mm^2^) as described earlier. The 208F*src3* cells were cultured for 94 h in the absence (the negative control) or presence of 1000 208F cells/mm^2^ either sham- or 0.5 Gy γ-irradiated.

**Figure 3:**
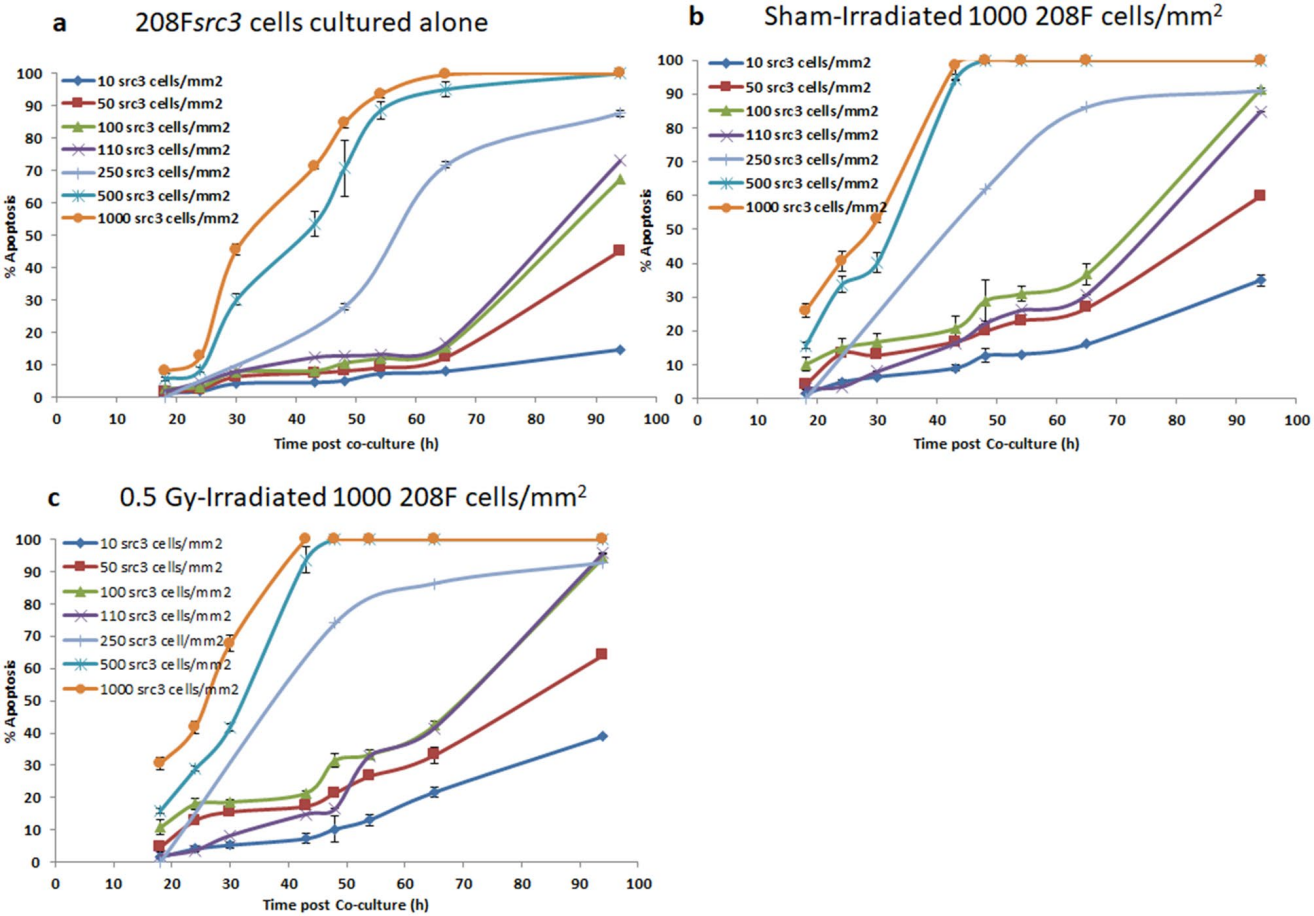
1000 cells/mm^2^ 208F cells and varying density of 208F*src3* cells. Apoptosis induction in transformed 208F*src3* cells seeded at seeding densities of 10, 50, 100,110, 250, 500 and 1000 cells/mm^2^ cultured in (**a**) the absence or presence of (**b**) sham- or (**c**) 0.5 Gy γ-irradiated non-transformed 208F cells/mm^2^ at a seeding density of 1000 cells/mm^2^. Each data point is a mean value derived from three independent experiments. Error bars represent the standard deviation derived from three independent experiments.

**Figure 4:**
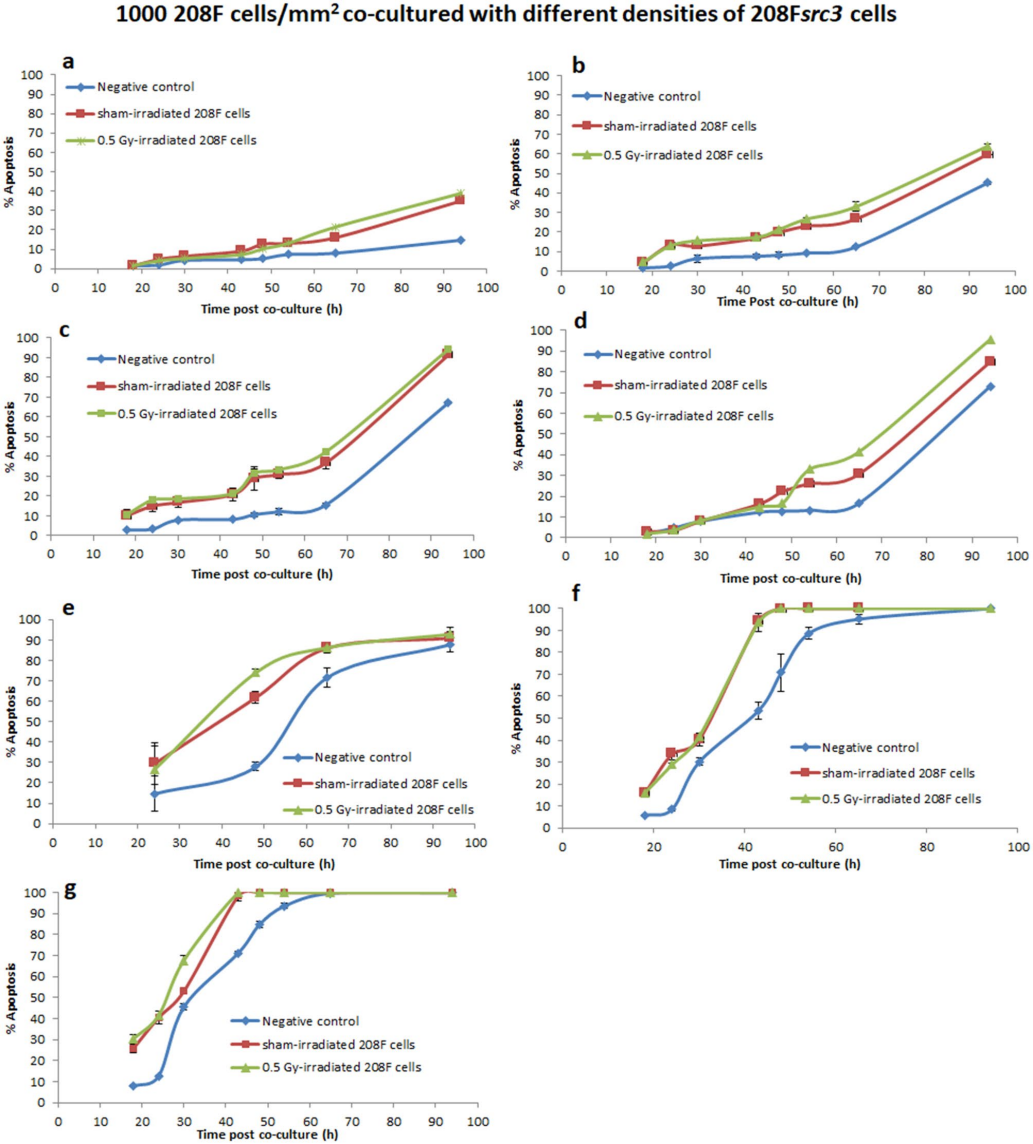
High density 208F cells and varying density of 208F*src3* cells. Apoptosis induction in transformed 208F*src3* cells seeded at densities of (**a**) 10, (**b**) 50, (**c**) 100, (**d**) 110, (**e**) 250, (**f**) 500 and (**g**) 1000 cells/mm^2^ cultured in the absence or presence of sham- or 0.5 Gy γ-irradiated non-transformed 208F cells at a seeding density of 1000 cells/mm^2^. Each data point is a mean value derived from three independent experiments. Error bars represent the standard deviation derived from three independent experiments.

The data in Figs. 3 and 4 show that co-culture of 208F*src3* cells seeded at a seeding density of 10 cells/mm^2^ with either sham- or 0.5 Gy γ-irradiated 208F cells, seeded at a density of 1000 cells/mm^2^, did not induce a significant IIA response in the 208F*src3* cells until 48 h post co-culture, at which the IIA response of the sham- and 0.5 Gy γ-irradiated groups were significantly higher than that of the negative control group (*p* < 0.05). Thereafter, the IIA response shows an increase above that of the negative control group. At 94 h post co-culture, although the IIA responses in the sham- and 0.5 Gy γ-irradiated groups are significantly (*p* < 0.05) higher than that of the negative control group, the slight differences observed between the sham- and 0.5 Gy γ-irradiated groups are not significant (Figs. 3b,c, 4a). Co-culture of 208F*src3* cells seeded at densities of 50 and 100 cells/mm^2^ with sham-irradiated 208F cells resulted in significant increase in the IIA response as compared with that of the negative control (*p* < 0.05). γ-irradiation of 208F cells shows an enhancement in the IIA response, as compared with the sham-irradiated group, only at the time points of 65 h and 94 h (*p* < 0.05) for 208F*src3* cells at a seeding density of 50 cells/mm^2^. However, for 208F*src3* cells at a seeding density of 100 cells/mm^2^, radiation-induced IIA enhancement was significant at early time points of 54 h and 65 h (*p* < 0.05), whereas no enhancement was recorded at the late time point of 94 h(Figs. 3b,c, 4b).

When 208F cells, either sham- or 0.5 Gy γ-irradiated, were co-cultured with 208F*src3* cells at seeding density of 110 cells/mm^2^ (Fig. 4d), the IIA response was evoked 48 h post co-culture with a further increase at later time points up to 94 h, when the level of induction of apoptosis in 208F*src3* cells was 73%, 84% and 95% for the negative control, sham- and 0.5 Gy γ-irradiated groups, respectively.

Co-culture of sham-irradiated 208F cells with 208F*src3* cells seeded at 250 cells/mm^2^ leads to no significant difference in the level of IIA response at the early 24 h and the late 94 h time points, when compared with the negative control. However, at the intermediate time points, 48 h and 65 h, the IIA response was significantly higher than that of negative control (*p* < 0.05). Irradiation of 208F cells prior to co-culture did result in a significant enhancement in the IIA response at the 48 h time point only (*p* < 0.05), after which the IIA response saturates for both sham and 0.5 Gy γ-irradiated groups.

On the other hand, for seeding densities of 500 and 1000 cells/mm^2^, IIA responses show significant increases 18 h post co-culture (*p* < 0.05), with no significant difference between the irradiated and sham-irradiated groups. Additionally, the overall kinetics of the IIA response in both sham- and 0.5 Gy γ-irradiated groups were shifted towards earlier time points as compared with the negative control group (Fig. 4e–g).

### IIA response in 208F*src3 *cells following co-culture with varying seeding densities of 208F cells either sham or 0.5 Gy γ-irradiated

To investigate the variation in IIA response in 208F*src3* as a function of the seeding density of co-cultured 208F cells, 208F*src3* cells seeded at 110 cells/mm^2^ were cultured for 94 h in the absence (negative control group) or presence of 208F cells seeded at 100, 500, 1000 and 5000 cells/mm^2^, and either sham- or 0.5 Gy γ-irradiated.

**Figure 5:**
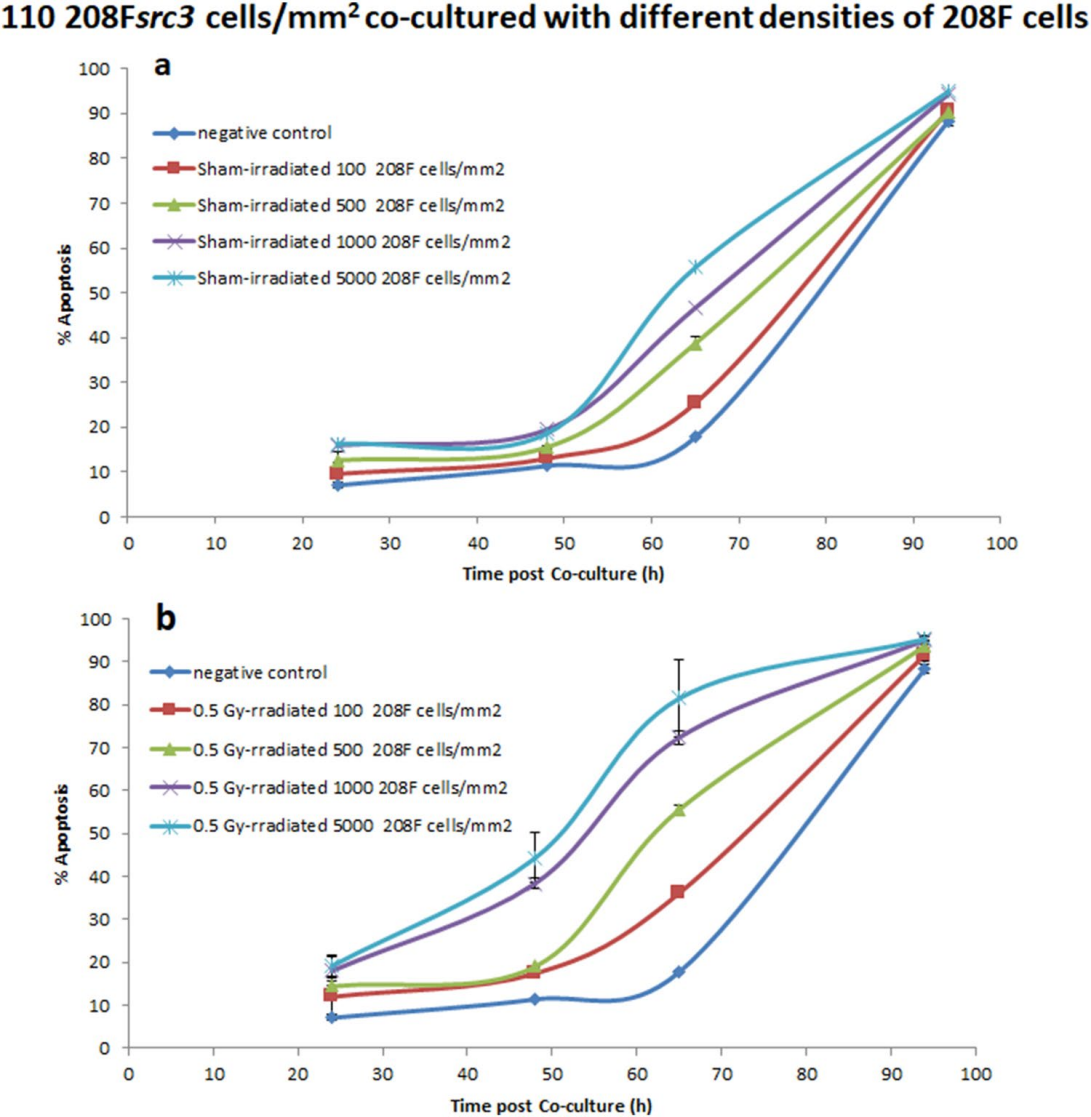
110 cells/mm^2^ 208F*src3* cells and varying density of 208F cells. Apoptosis induction in transformed 208F*src3* cells seeded at a seeding density of 110 cells/mm^2^ cultured in the absence (negative control) or presence of 208F cells seeded at densities of 100, 500, 1000 and 5000 cells/mm^2^ either (**a**) sham- or (**b**) 0.5 Gy γ-irradiated. Each data point is a mean value derived from three independent experiments. Error bars represent the standard deviation derived from three independent experiments.

**Figure 6:**
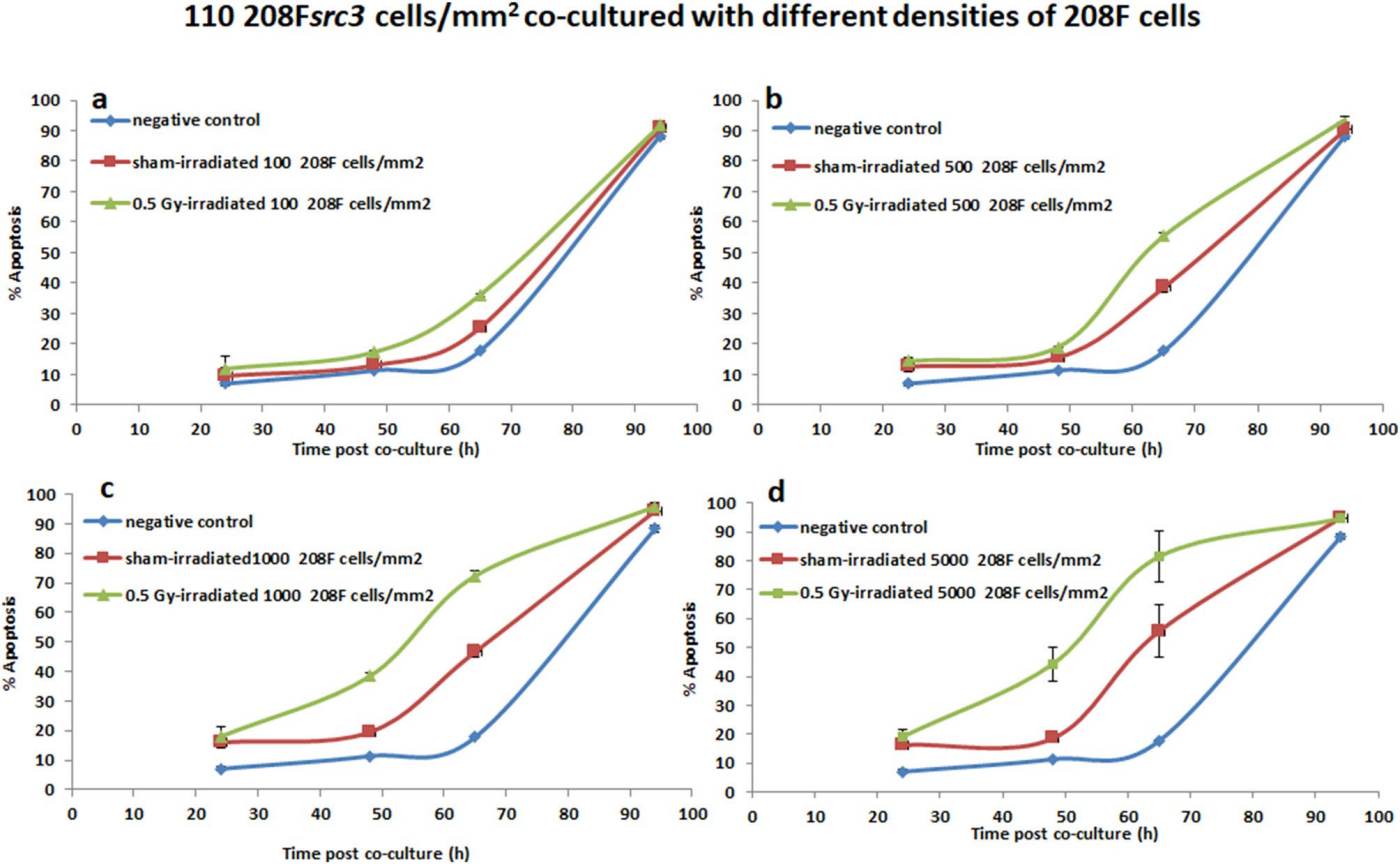
Varying 208F cells density and medium density 208F*src3* cells. Cell density dependence apoptosis induction in transformed 208F*src3* cells seeded at a seeding density of 110 cells/mm^2^ cultured in the absence (negative control) or presence of 208F cells seeded at densities (**a**) 100, (**b**) 500, (**c**) 1000 and (**d**) 5000 cells/mm^2^ and either sham- or γ-irradiated with a dose of 0.5 Gy. Each data point is a mean value derived from three independent experiments. Error bars represent the standard deviation derived from three independent experiments.

The data in Fig. 5a show that co-culture of the 208F cells with 208F*src3* cells leads to induction of IIA response in 208F*src3* cells, with the IIA response increasing as the initial seeding density of the 208F cells increases. Irradiation of 208F cells with 0.5 Gy γ-rays further boosts the IIA response and leads to a shift in the IIA response towards earlier times (Fig. 5b). Enhancement of IIA response following 0.5 Gy γ-irradiation of 208F cells seeded at 100 and 500 cells/mm^2^was significant from 48 h post co-culture (Fig. 6a,b). However, for the higher densities of 1000 and 5000 cells/mm^2^, the radiation-induced enhancement of IIA response was shifted towards earlier time point of 24 h post-culture (Fig. 6c,d).

## Discussion

This study demonstrates the pivotal role of the cell density of either transformed or non-transformed cells in modulating both autocrine destruction (AD) and intercellular induction of apoptosis (IIA). AD and IIA are multi-stage signaling mechanisms involving orchestrated ROS/RNS and cytokine signaling^3,18,19^. The kinetics and magnitudes of AD or IIA responses are correlated to the abundance of the signals present in the system, hence depend on the cell density or the number of the cells in the system.

AD kinetics in transformed cells shows dependency on both cell density and culture time (Fig. 2a). A remarkable increase in AD response in transformed cells was observed as a consequence of increasing the cell density. For the higher seeding densities of 250, 500 and 1000 cell/mm^2^, AD was elicited as early as 25–30 h post-culture with saturation level reached for the two higher densities at 65 h. On the contrary, medium/low densities show background levels of AD response up to 50–65 h followed by an increase in response with time but saturation levels were seen up to 94 h. The increase in AD in transformed cells with seeding density is expected, based on our previous findings^1,2,15,20^, since increasing the density of transformed cells is expected to results in higher concentrations of superoxide anions (O_2_^−·^) formed in close vicinity of the cell membrane, consistent with an increase in the formation of hydrogen peroxide (H_2_O_2_), through the dismutation of O_2_^−·^. Simultaneously, since the transformed cells in this case are the source of peroxidase (POD) and TGF-β^1,21^, increasing the cell density would cause more peroxidase (POD) and TGF-β release, resulting in an overall enhancement of apoptosis induced by the POD/HOCl pathway (Supplementary Figs. 2 and 3).

Increasing the seeding density of transformed cells reduces the time required to elicit the AD response, which was readily seen for the high densities of 250, 500 and 1000 cell/mm^2^. This is related to the time taken for these actively growing cells to reach the required cell densities. The cells at high densities will not only have sufficient signalling molecules at earlier time than that for low densities but also the local concentration of superoxide anions (O_2_^−·^) will be sufficiently high for the dismutation reaction to occur faster. Although none of the densities, apart from the 500 and 1000 cell/mm^2^, reach the saturation level during the time course of the experiments. The other densities may reach the saturation level at later time points. However at theses time points the densities will be far greater than the initial density seeded. This again highlights the role of seeding density in shifting the onset of AD towards earlier times.

Our previous data show that co-culture of transformed cells with non-transformed cells in the absence of external stimulus (ionizing radiation or exogenous TGF-β) leads to augmenting apoptosis induction in transformed cells as a result of the intercellular signalling between non-transformed and transformed cells, known as IIA response, previously described^1^. In the absence of external stimulus, the NO^·^/ONOO^−^ pathway dominates the IIA signalling since NO^·^ is sufficiently abundant to outcompete endogenous levels of superoxide dismutase for O_2_^−·^^22^. The kinetics of the IIA response is dependent on the abundance of the signaling molecules present, hence on cell densities of either cell type^11,19^.

Our results show that when 100 cells/mm^2^ of non-transformed cells were used as effector cells (Fig. 2), no significant IIA response was detected for all densities used for transformed cells apart from the medium densities of 100 and 110 cells/mm^2^,where IIA response was only detectable and distinguishable from the AD response 54 h post co-culture. However, the rise in IIA response is slightly earlier (~ 40 h) when high densities of irradiated non-transformed cells are used. For IIA response to provoke sufficient amount of signalling molecules including non-transformed cells-derived NO^·^, transformed and non-transformed cells-derived TGF-β as well as transformed cells-derived O_2_^−·^ should be present. Since a fixed density of non-transformed cells (100 cells/mm^2^) is used, we assume that the amount of NO^·^ and non-transformed cells-derived TGF-β will remain the same, whilst the levels of O_2_^−·^ and transformed cells-derived TGF-β will vary substantially between the very low and high densities of transformed cells.

Despite the fact that no obvious IIA response was detected for both very low and high densities of transformed cells, the reason behind this may be significantly different. A possible explanation is that at very low densities of transformed cells (10 and 50 cells/mm^2^), too few O_2_^−·^ molecules will be available to produce apoptosis inducing peroxynitrite (ONOO^−^) through interaction with NO^·^ released by non-transformed cells. While, at high densities of transformed cells (250, 500 and 1000 cells/mm^2^) enhanced levels of O_2_^−·^ and peroxidase (POD) are produced by the transformed cells themselves. Thus apoptosis in the transformed cells will overwhelmingly occur by the POD/HOCl pathway of AD, since any NO^·^ produced by the neighbour non-transformed cells will not significantly add to the overall effect. As a consequence, apoptosis in transformed cells will be dominated by AD.

For intermediate densities of transformed cells, AD signalling dominates the effect up to 45 h, as evident from comparing the co-culture data with the negative control group,. It is proposed that at early time points the consumption of O_2_^-·^ present may be dominated by the POD/HOCl pathway of AD, thus reducing its consumption by NO^·^/ONOO^−^ pathway of IIA response. At later time points, 54 h and beyond, high levels of O_2_^−·^ are available for consumption by both POD/HOCl pathway of AD and NO^·^/ONOO^−^ pathway of IIA along with increased IIA signaling due to the increasing density of non-transformed cells as they grow and divide. These considerations however emphasize the importance of the balance between the different signaling pathways. It is worth noting that at later time points more transformed cells are present in the system, hence greater levels of O_2_^−·^ are produced. Co-culture of transformed cells with higher densities of sham-irradiated non-transformed cells (500, 1000 and 5000 cells/mm^2^) result in a shift in the kinetics of IIA response towards earlier time points. Increasing the density of non-transformed cells increases the amount of NO^·^ released and therefore the amount of apoptosis inducing peroxinitrite produced through the NO^·^/ONOO^−^ pathway of IIA. These assumptions are supported by our previous findings using ebeselen as a specific scavenger for ONOO^−^^20^ (Supplementary Fig. 4).

These data are paradoxical to the prediction of Kundrat et al.^17^ who suggested a bell shaped (U-shaped) dependence of IIA response as a function of seeding density of transformed cells, in a modelled system very similar to that used in this work. The modelled system assumed that the non-transformed cells were pre-treated with TGF-β, which has previously been shown to give a very similar response to that for irradiated cells^2^. Starting at low transformed seeding densities, apoptosis was predicted to decrease with increasing seeding density, followed by a subsequent increase as seeding densities increase above ~ 100 cells/mm^2^. As the seeding density of non-transformed ‘effector’ cells increases, the decrease and subsequent rise in apoptosis dependence is predicted to get sharper and narrower. Although the experimental data presented here covered a very similar range of seeding densities (10–1000 transformed cells/mm^2^ at either 100 or 1000 non-transformed cells/mm^2^) no evidence of a reduction in apoptosis was observed.

We and others have previously shown that irradiation of non-transformed cells prior to co-culture with transformed cells amplifies the IIA response by increasing the release of NO^·^ and hence augmenting the NO^·^/ONOO^−^ as well as stimulating the production of POD by non-transformed cells^2,4,5,23^. Thereby, irradiation of non-transformed cells not only enhances the NO^·^/ONOO^−^ pathway but also elicits the POD/HOCl pathway which contributes to the IIA signaling^1^. Additionally, irradiation of transformed cells is reported to lead to increased levels of O_2_^−·^^24^, POD and NO^·^^15^ production. It is worth noting that transformed cells upon stimulation by TGF-β treatment or radiation can also acquire the effector function and induce apoptosis in co-cultured transformed cells and suggests that radiation stimulation is a result of an increase in TGF-β production (or activation) by the irradiated cells^25^. While these experiments were performed using a γ-ray dose of 0.5 Gy, previous studies have shown that doses as low as 2 mGy may also result in an enhancement of the IIA response, with the effect saturating at a dose of 50 mGy or above^5^.

Our results show that the enhancement of IIA response by radiation is dependent on the density of the irradiated non-transformed cells and the non-irradiated transformed cells. Even though irradiation promotes the release of signaling molecules from non-transformed cells, sufficient concentration of O_2_^−·^ is still needed for the onset of IIA to be seen. Therefore, the density of transformed cells and/or time for transformed cells to reach sufficient numbers is needed to produce sufficient levels of O_2_^−·^ for both AD and IIA signaling. Hypothetically, at low densities of irradiated non-transformed cells, the onset of IIA signalling requires the build up of signaling molecules to be able to detect IIA response. This is in agreement with previous data reported that the local density of transformed cells rather than absolute number cells is important for the target function of transformed cells, while sufficient number rather than high local density is important for the effector function, even if the effector cells were the stimulated transformed cells^25^.

Increasing the density of non-transformed cells enhances the IIA response as a result of increasing the amount of signalling molecules involved mainly in the NO^·^/ONOO^−^ pathway (dominating the IIA signalling in absence of radiation). Irradiation of non-transformed cells perturbs the whole signalling mechanism by enhancing the NO^·^/ONOO^−^ pathway and stimulating the production of peroxidase involved in the POD/HOCl pathway. IIA response is based on the balance between the different signalling pathways involved. Increasing the activity of one of the pathways may influence the other pathways. Though, this may or may not be translated into an apparent effect on the IIA response. The data presented support previous experimental findings showing that exposure to ionizing radiation results in the enhanced removal of pre-cancerous cells as a result of IIA. However, the degree of enhancement and associated kinetics is dependent on the relative densities of the transformed and non-transformed cells. Additionally, the data provided no evidence that exposure to radiation could potentially result in an increase in the survival of these pre-cancerous cells as a result of a decrease in IIA for the wide range of seeding densities explored.

## Methods

### Cell lines and cell cultures

Non-transformed (208F) and v-src-transformed (208F*src3*) rat fibroblast cell lines described before^7,23^ were used in the passage range 18–30. Cells were grown in Eagle’s minimum essential medium (MEM) (Sigma, UK) supplemented with 5% FBS (Sigma, UK), 2 mM l-glutamine and 50 mg/ml penicillin–streptomycin in a penicillin–streptomycin-glutamine solution (Gibco, UK). The cultures were incubated at 37 °C in a 5% CO_2_/air gassed incubator.

### Calculation of seeding densities

For the purpose of this study, different seeding densities of the 208F and 208F*src3* cells were used to seed the required initial number of cells (Table 1). Cells grown in cell culture flasks were processed by removing the medium then washing with 10 ml PBS. Cells were then trypsinized with 5 ml trypsin–EDTA and transferred into 15 ml medium in falcon tube before centrifugation at 650 g for 5 min. The supernatant was then removed and the cell pellet was resuspended in 5 ml of medium. The cell density was determined using a counting chamber (Webber Scientific International, UK) and the cells subsequently diluted in medium to give the required densities listed in Table 1.

**Table 1:** Transformed and non-transformed cells seeding densities.

Cells seeding density (cells/ml)		Respective initial number of cells seeded per unit area (cells/mm^2^)	Designation of the seeding density
Transformed 208F*src3* cells (6 well plate)	9000	10	Low density
	45,000	50	
	90,000	100	Medium density
	100,000	110	
	227,000	250	High density
	454,000	500	
	909,000	1000	
Non-transformed 208F cells (insert)	46,000	100	Medium density
	231,000	500	High density
	46,463,000	1000	
	2,270,000	5000	Very high density

The calculation of the seeding densities of both cell lines were carried out based on the surface area of the containing well. 208F cells were seeded in 1 ml of medium in cell culture plastic inserts with transparent base membrane of pore diameter: 0.4 µm for 6-well plates (Thincert, Greiner-bio one, UK). The inner surface area of the insert is 462.4 mm^2^. The seeding density 110 cells/mm^2^ is included for compatibility with previous experiments^1,2,5,23^.

The transformed208F*src3* cells were seeded in 2 ml of medium in 6-well plates (Greiner-bio one, UK), where the inner surface area of each well is 900 mm^2^. Upon co-culture, one more ml of medium was added to the transformed cells.

Transformed 208F*src3* cells were seeded in 6-well plates either alone or in co-culture with non-transformed 208F cells in 3 ml of medium in total. For co-culture experiments, both cell lines were initially cultured separately to allow cells to attach, with the inserts containing 208F cells placed in empty 6-well plates. Then, 5 h later, 208F cells were either sham or 0.5 Gy-irradiated and then immediately co-cultured with the 208F*src3* cells for 94 h using the co-culture set up previously described^1,2^.

### Experimental set up

Three sets of experiments were performed with each set performed in triplicate. In the first two sets, 208F*src3* cells seeded at seeding densities 10, 50, 100,110, 250, 500 or 1000 cells/mm^2^ were co-cultured with 100 or 1000 208F cells/mm^2^, either sham- or 0.5 Gy γ-irradiated, or cultured alone. To serve as negative control, when 208F*src3* cells are cultured alone, a blank insert with medium only was used instead of the inserts containing 208F cells. In the third set, 110 208F*src3* cells/mm^2^ were co-cultured with, either sham- or 0.5 Gy γ-irradiated 208F cells seeded at seeding densities 100, 500, 1000 or 5000 cells/mm^2^. After seeding, both cell lines were left to attach for 5 h.

### γ-Irradiation

Five hours after cells seeding, 208F cells were γ-irradiated in plastic inserts supported in empty 6-well plates at room temperature using a GSR D1 ^137^Cs γ-irradiator (Gamma-Service Medical GmbH, Leipzig, Germany) at the Oncology Department, University of Oxford at a dose rate of 1.76 Gy/min.

### Quantification of apoptosis induction in 208Fsrc3 cells

Apoptosis induced in 208F*src3* cells was quantified using the morphological features of cells undergoing apoptosis of chromatin condensation and fragmentation as previously described^2,21,23^. 208F*src3* cells are easily identified, as they were cultured in the wells of the 6-well plates, while the non-transformed 208F cells were cultured in inserts. Imaging of the cells was carried out using a Nikon TS-100F (Nikon, UK) inverted phase-contrast microscope. The percentage of apoptotic 208F*src3* cells was determined by scoring at least 200 cells from random positions in each well. Three wells were scored for each point and the average was calculated. Scoring was carried out at different time points up to 94 h post co-culture. Three independent repeats of each experiment were performed. The error bars in the figures represent the standard deviation derived from three independent experiments.

### Statistical analysis

Each data point represents the average from three independent experiments. The standard deviation was calculated and shown in the figures. The *p*-values were calculated using the Microsoft Excel statistical tool two-way ANOVA with replication. Difference was noted significant if the p-value < 0.05.

## Supplementary Information

Supplementary Information.

## Data Availability

All data generated or analysed during this study are included in this published article.
