# Supplementary material for: Influence of ionizing radiation and cell density on the kinetics of autocrine destruction and intercellular induction of apoptosis in precancerous cells
Source: Sci Rep. 2022 May 3;12:7150. doi: 10.1038/s41598-022-11253-1 (PMC9065116; doi:10.1038/s41598-022-11253-1)
Supplement: Supplementary file 1 — Supplementary Information. [file 41598_2022_11253_MOESM1_ESM.pdf]

Influence of ionizing radiation and cell density on the kinetics of autocrine destruction and intercellular induction of apoptosis in precancerous cells

Abdelrazek. B. Abdelrazzak[1*], Peter O'Neill[2] and Mark A. Hill[2].

[1]Spectroscopy Department, Physics Research Institute, National Research Centre, Cairo, 12622, Egypt.

[2]MRC Oxford Institute for Radiation Oncology, University of Oxford, Gray Laboratories, ORCRB Roosevelt Drive, Oxford OX3 7DQ, UK.

Supplementary Data

IIA response in 208Fsrc3cells seeded at varying seeding densities in absence or presence of 100 cells/mm² of 208F cells either sham or 0.5 Gy γ-irradiated.

When 100 208F cells/mm^2 were co-cultured with varying densities of 208Fsrc3 cells, no difference in apoptosis induction was seen between the very low seeding densities (10 and 50 cells/mm^2) of 208Fsrc3 cells, apart from a slight difference at 94 h (see Supplementary Figure 1a & b). However, for the medium seeding densities (100 and 110 cells/mm^2), enhancement in IIA response was seen only at 54 h and beyond (Supplementary Figure 1 c & d). Co-culture of 100 208F cells/mm^2 with 250 208F*src3* cells/mm^2 did not result in increase in apoptosis induction in 208F*src3* cells (Supplementary Figure 1 e). On the other hand, co-culture of 100 208F cells/mm^2 with high densities of 208Fsrc3 cells (500 and 1000 cells/mm^2) results in shift in the time point of the maximum apoptosis saturation level reached (the plateau level) towards an earlier time point of 54 h (Supplementary Figure 1 f & g) as compared with the negative control group (65h), with no significant difference at earlier time points.

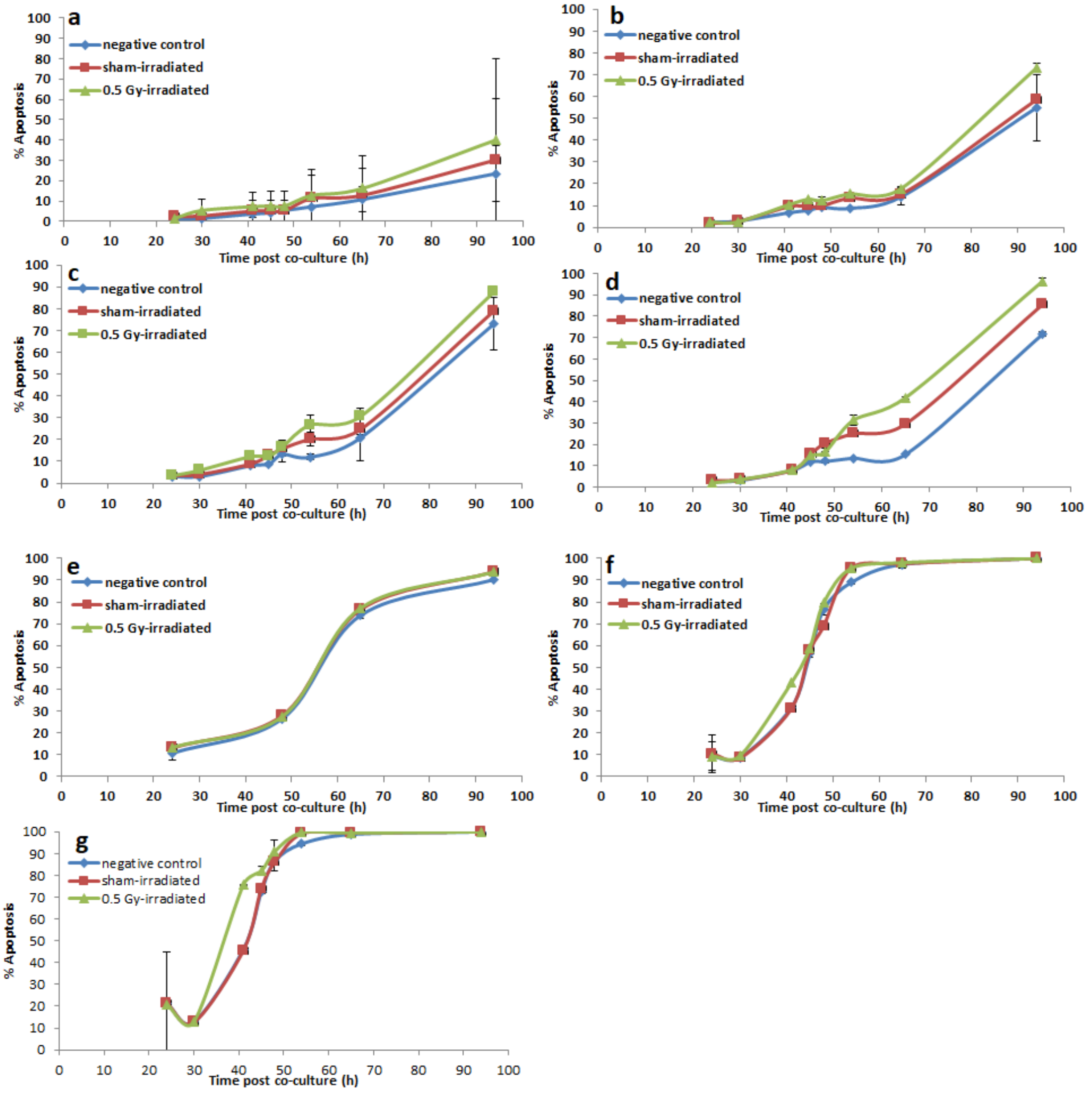

Supplementary Figure 1: **Apoptosis induction in transformed 208F*src3* cells at varying seeding densities**. Apoptosis induction in transformed 208F*src3* cells seeded at seeding densities of (a) 10, (b) 50, (c) 100, (d) 110, (e) 250, (f) 500 and (g) 1000 cells/mm^2 and cultured in the absence or presence of sham- or 0.5 Gy γ-irradiated non-transformed 208F cells at a seeding density of 100 cells/mm^2.

The signalling pathways involved in autocrine destruction and intercellular induction of apoptosis under varying cell densities

The involvement of the POD/HOCl and NO/ONOO- signaling pathways in AD response under the condition of varying cell densities has been verified by our group [20]. To investigate the dependence of the signaling pathway involved in autocrine destruction (AD) on the 208Fsrc3 cell density, varying cell densities of 208Fsrc3 cells were cultured for 64 h in the presence or absence of Taurine (HOCl scavenger) and Catalase (H_2O_2 scavenger), both known to block the POD/HOCl signaling pathway. The data in Supplementary Figure 2 show that increasing the cell density of 208src3 cells increases the percentage of transformed 208Fsrc3 cells undergoing apoptosis. Addition of the H_2O_2 scavenger, catalase, or HOCl scavenger taurine significantly reduces the percentage of cells undergoing apoptosis to the same level for all cell densities used. These data show that increasing the cell density augmented the autocrine response through the same pathway (POD/HOCl pathway) and that changing the cell density did not alter the nature of the signals involved in AD but only affected the kinetics of the response. These data are also in agreement with the previously reported data by Von Eynatten and Bauer (2001)[19] , where in part of their study, 208F*src3* cells were seeded at density of 200 cells/mm^2 (close to one of the high densities we used in the manuscript) in absence of non-transformed 208F cells. Their data also suggested the POD/HOCl pathway as the pathway responsible for apoptosis induction in 208Fsrc3 cells cultured in absence of 208F cells.

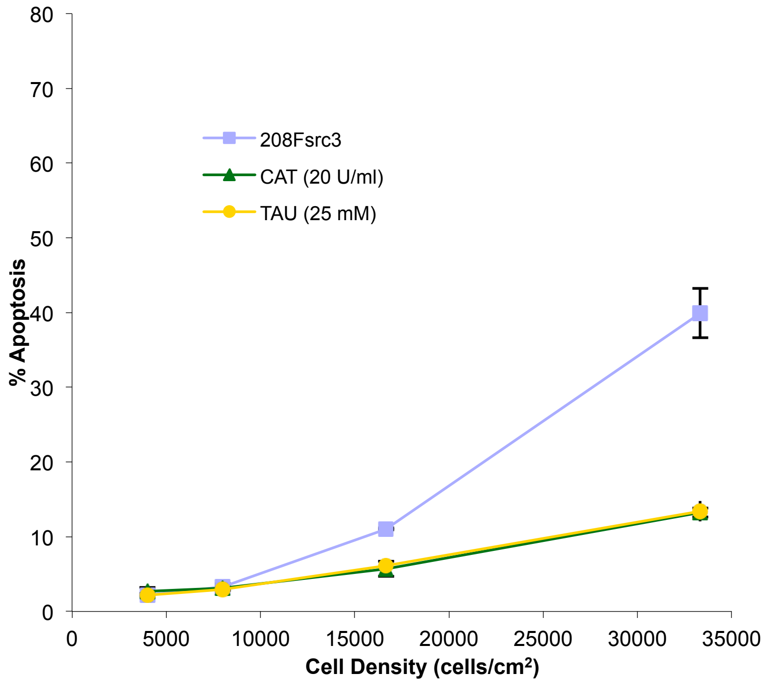

Supplementary Figure 2: Signaling pathways involved in AD. The effect of HOCl scavenger catalase (20 U/ml) and H_2O_2 scavenger taurine (25 mM) on apoptosis induction in 208F*src3* cells seeded at varying cell densities [20].

The effect of ionizing radiation on the AD signaling pathway

The effect of ionizing radiation on the AD signaling pathway for varying cell densities of 208F*src3* cells was studied (Supplementary figure 3). Varying cell densities of 208F*src3* cells, either sham- or 0.5 Gy-irradiated were cultured in the absence or presence of H_2O_2 scavenger, catalase, or the HOCl scavenger taurine. The data in Supplementary figure 3 show that 2 Gy of γ-rays leads to an increase in the percentage of 208F*src3* cells undergoing apoptosis at all cell densities studied. The involvement of the POD/HOCl signalling was again confirmed using catalase and taurine since both catalase and taurine significantly reduce the percentage of cells undergoing apoptosis following γ- irradiation with 2 Gy.

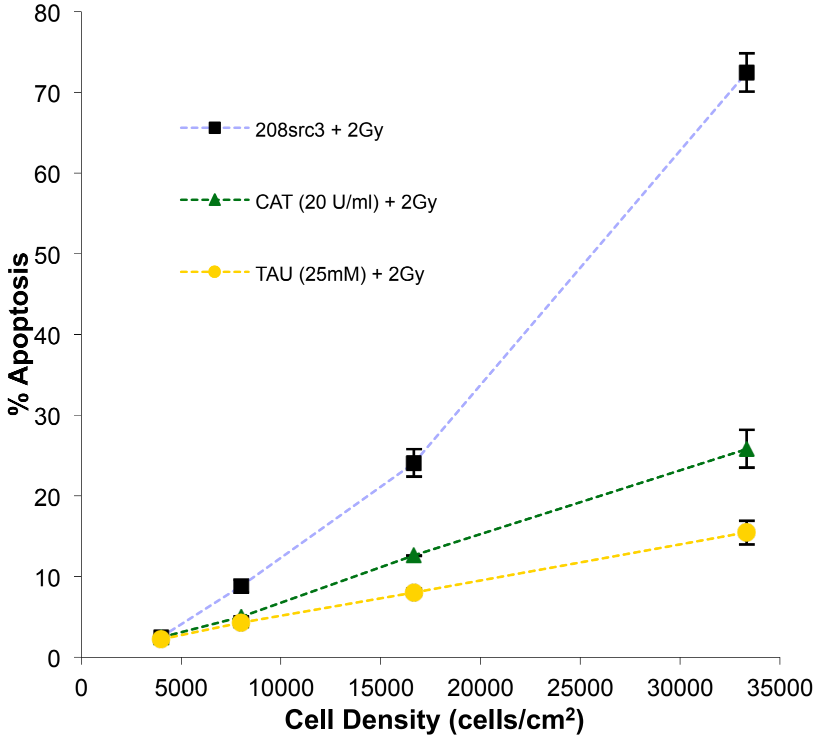

Supplementary Figure 3: The effect of cells density and ionizing radiation on AD signaling pathway. The effect of treatment of 208F*src3* cells seeded at varying cell densities with either catalase (20 U/ml) or taurine (25mM) following irradiation with 2 Gy γ-rays [20].

The effect of cell density as well as ionizing radiation on the IIA signaling pathways

To investigate the effect of cell density as well as ionizing radiation on the IIA signaling pathways, 208F*src3* cells were co-cultured for 68h in the presence or absence of 25 mM of taurine, HOCl scavenger, (to block the POD/HOCl pathway), or 4μM ebselen, an $ONOO^-$ scavenger (to block the $NO^\bullet/ONOO^-$ pathway) with 208F cells seeded at a high seeding density of ~500 cells/mm^2, either sham- or irradiated with 2 Gy γ-rays. The data in Supplementary figure 4 clearly indicate the involvement of both POD/HOCl and $NO^\bullet/ONOO^-$ pathways in inducing apoptosis in transformed 20Fsrc3 cells. These finings are in agreement with the previously obtained data by Herdner and colleagues (2000) [11], where they also confirmed, using relevant scavengers the involvement of both POD/HOCl and $NO^\bullet/ONOO^-$ pathways in IIA response using low (10 cells/mm^2) and high (200 cells/mm2) of 208F*src3* cells as target cells.

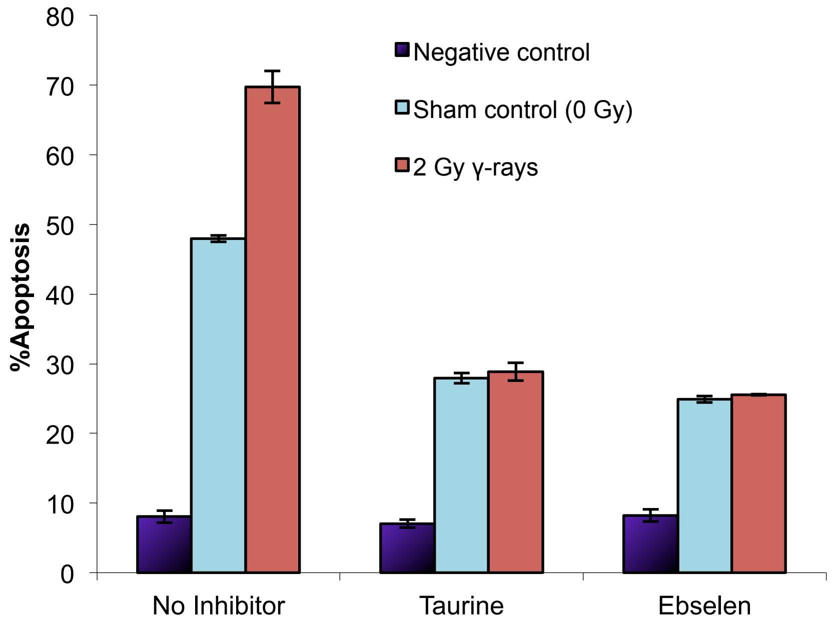

Supplementary Figure 4: Signaling pathways involved in IIA response. Percentage apoptosis scored in non-irradiated transformed cells co-cultured for 68h in the presence or absence of taurine, or ebselen with 208F cells (seeded at ~500 cells/mm^2) irradiated with 2 Gy γ-rays [20].

These data clearly indicate the involvement of both the POD/HOCl and $NO^{\bullet}$/$ONOO^-$ pathways in the IIA signaling when high density of 208F cells was co-cultured with either low or high cell densities of 208Fsrc3 cells.
